# Supplementary material for: Sequential Organ Failure Assessment Outperforms Quantitative Chest CT Imaging Parameters for Mortality Prediction in COVID-19 ARDS
Source: Diagnostics (Basel). 2021 Dec 22;12(1):10. doi: 10.3390/diagnostics12010010 (PMC8775048; doi:10.3390/diagnostics12010010)
Supplement: Supplementary file 1 [file diagnostics-12-00010-s001.zip › diagnostics-1515793-supplementary.pdf]

diagnostics

Supplemental Material

Sequential Organ Failure Assessment Outperforms Quantitative Chest CT Imaging Parameters for Mortality Prediction in COVID-19 ARDS

Table S1. COVID-19 ICU Patients comparing Survivors vs. Non-Survivors.

	Survivors (n = 53)		Non-Survivors (n = 36)		p value
**Comparison of Patient Characteristics**					
Age	62	(52-70)	68	(59-81)	**p = 0.005**
Male Sex	40	(75.5%)	30	(83.3%)	p = 0.374
BMI	28	(25-33)	27	(25-32)	p = 0.482
**ARDS Type on Admission**					p = 0.634
Mild	17	(34.7%)	7	(21.9%)	
Moderate	22	(44.9%)	17	(53.1%)	
Severe	8	(16.3%)	7	(21.9%)	
No ARDS on Admission	2	(4.1%)	1	(3.1%)	
**Patient Data during ICU Stay**					
Days on ICU (including external ICUs)	13.7	(7.9-23.9)	17.3	(6.4-26.6)	p = 0.789
Number of Patients on Mechanical Ventilation	38	(71.7%)	33	(91.7%)	**p = 0.021**
Hours on Ventilator	163.4	(0.0-359.0)	339.8	(115.6-590.1)	**p = 0.038**
Number of Patients on NIV	35	(66.0%)	15	(41.7%)	**p = 0.023**
Hours on NIV	3.2	(0.0-31.0)	0.0	(0.0-11.3)	**p = 0.044**
Number of Patients with HDF	13	(24.5%)	26	(72.2%)	**p < 0.001**
Number of Patients with ECMO	2	(3.8%)	12	(33.3%)	**p < 0.001**
SOFA mean*	7.1	(4.6-8.4)	13.8	(11.4-16.2)	**p < 0.001**
SOFA max*	11	(7-13)	18	(14-22)	**p < 0.001**
SOFA on Admission*	7	(4-10)	11	(8-14)	**p < 0.001**
Oxygenation Index on Admission**	184	(120-247)	144	(105-199)	p = 0.055
Lactate on Admission	1.2	(0.9-1.7)	1.5	(1.1-1.8)	p = 0.092
**Imaging Data**					
CT Severity Score on Admission***	15	(11-19)	16	(10-21)	p = 0.608
CT Percentage of Lung Involvement on Admission***	30	(17-53)	34	(18-67)	p = 0.578
CT Severity Score on SOFA max****	17	(14-19)	18	(14-21)	p = 0.616
CT Percentage of Lung Involvement on SOFA max****	49	(31-56)	47	(24-64)	p = 0.969
Pulmonary Artery to Ascending Aorta Ratio	0.87	(0.79-0.95)	0.85	(0.77-0.91)	p = 0.504
**Comorbidities**					
Hypertension	29	(54.7%)	25	(69.4%)	p = 0.163
Diabetes	13	(24.5%)	17	(47.2%)	**p = 0.026**
Heart Disease	11	(20.8%)	19	(52.8%)	**p = 0.002**

Pulmonary Disease	9	(17.0%)	7	(19.4%)	p = 0.766
Malignancy	3	(5.8%)	6	(16.7)	p = 0.097
Immunosuppression	2	(3.8%)	5	(13.9%)	p = 0.082

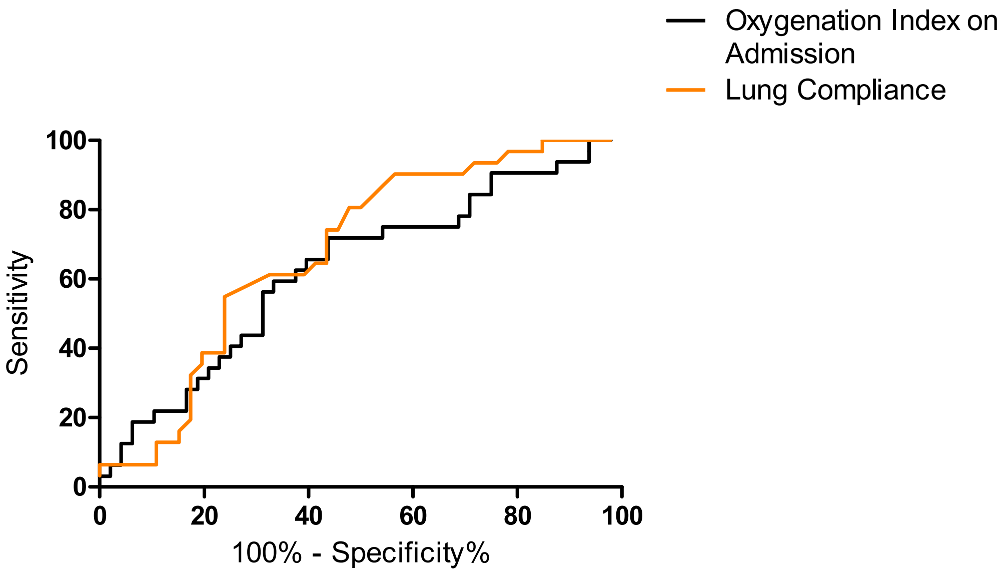

Figure S1. ROC curves for ox-ygenation index on ICU admission and lung compliance on ICU admission.
